# Genome-wide association studies and transcriptome analysis reveal novel genes associated with freezing tolerance in rapeseed (*Brassica napus L.*)

**DOI:** 10.1371/journal.pone.0322547

**Published:** 2025-05-27

**Authors:** Guoqiang Zheng, Zigang Liu, Lixi Jiang, Qi Yang, Jiaping Wei, Zefeng Wu, Junmei Cui, Xiaoyun Dong, Xiaodong Cao, Xuezhen Yang, Ying Wang, Yongjie Gong, Ermei Sa, Xiaoxia Wang

**Affiliations:** 1 State Key Laboratory of Aridland Crop Science/College of Agronomy, Gansu Agricultural University, Lanzhou, Gansu, China; 2 College of agricultural & biotechnology, Zhejiang University, Hanzhou, Zhejiang, China; 3 Agricultural information center of Gansu Province, Lanzhou, Gansu, China; 4 Hybrid Rapeseed Research Center of Shaanxi Province, Yangling, Shaanxi, China; 5 Seed management station of Ping Liang, Pingliang, Gansu, China; South China Agricultural University, CHINA

## Abstract

Freezing stress is the main obstacle affecting the geographical distribution, growth, development, quality, and productivity of rapeseed (*Brassica napus*) in northern China. However, there is a little knowledge of rapeseed freezing tolerance mechanism. Here, 289 core germplasms collected from 36 countries were used to identify SNPs associated with freezing tolerance. We used RNA-seq data to narrow down the candidate genes identified by genome-wide association studies. The frequency distributions of phenotypic values and best linear unbiased estimates (BLUE) values for each trait conform to normal or approximately normal distributions, with good repeatability across various locations. The results showed that 594, 513, 7, and 45 SNPs were significantly associated with malondialdehyde, peroxidase, soluble protein, and relative electrolyte leakage, respectively. Based on these significantly associated SNPs, we identified 4,998 associated genes. Crossover analysis indicated that 73 genes were overlapped between GWAS and RNA-seq datasets, and 13 candidate genes involved in transmission and perception of freeze stress signals, lipid metabolism, reactive oxygen species (ROS) homeostasis, antifreeze proteins synthesis, and other metabolic processes. These results reveal novel genes associated with freezing tolerance in rapeseed, and provide a basis for further research and improvement of freezing tolerance in rapeseed.

## Introduction

Rapeseed is an important source of edible oil in many parts of the world. In China, rapeseed contains two major producing areas of winter rapeseed and spring rapeseed, and the northern boundary of the winter rapeseed producing area is generally 35° north latitude [1]. In winter, the lowest temperature in most winter rapeseed-producing areas is below 0 °C and lasts for a period of time, and the extreme low temperature is even lower than −15 °C [2,3]. Therefore, freezing tolerance is the most important and necessary characteristic of northern winter rapeseed cultivars. The freezing tolerance of rapeseed seedlings is a complex process affected by many factors, including environmental and genetic factors [4]. During production, freezing tolerance is also enhanced through winter soil covering or chemical spraying to reduce the impact of freezing stress on the yield and quality of rapeseed [5]. Due to the unpredictability of the climate, the effect is not remarkable, and it will increase the cost of planting. Therefore, identifying and characterizing freezing tolerance-related genes will help clarify the genetic mechanisms of freezing tolerance and will accelerate the breeding efficiency of freezing-tolerance cultivars in rapeseed.

Freezing tolerance is a complex quantitative trait, and numerous quantitative trait loci (QTL) for freezing tolerance have been detected in rapeseed through linkage mapping and genome-wide association studies (GWAS). A total of 16 QTLs were identified in 174 individual plant F2 and F2:3 populations [6]. Two major QTLs (*qOWRTA07* and *qOWRLA07*) were detected in two separate trials, with a single QTL explaining more than 10% of the phenotypic variation, and located between 21.4Mb and 23.4Mb on chromosome A7 (ZhongShuang11), which contains the gene encoding beta-1,3-glucanase (*BnAFP*). Overexpression of the beta-1,3-glucanase gene (*BraAFP*) results in enhanced freezing tolerance in transgenic Arabidopsis plants [7]. Meanwhile, the fragment from the –933 to –836 site away from the transcription start site of the BrAFP1 promoter drives gene-specific expression in leaves and stems of winter rapeseed *(Brassica rapa L.)* under cold induction [8]. In addition, several SNPs associated with freezing stress were identified in the GWAS analysis of *Brassica napus*, and related genes were involved in plant photosystem protection, cell membrane stability and RNA integrity, lipid and fatty acid metabolism, photosynthesis, flowering, ubiquitination, and cytochrome P450-related proteins [9–12].

GWAS is an efficient strategy to identify functional genes controlling agronomic or quality traits, and the major challenge is the accurate evaluation of traits. Previous studies have shown that relative electrolyte leakage (REL), malondialdehyde (MDA) content, peroxidase (POD) activity, and soluble protein (SP) levels can be used to indirectly assess the freezing tolerance in rapeseed under freezing stress [13,14]. Meanwhile, the field conditions of freezing stress in this study provides a strong condition for the success of the experiment, compared with indoor simulation experiment. The core germplasm used in this study was selected from a worldwide collection of 991 *Brassica napus* genetic materials originating from 39 countries, representing more than 97% of the SNPs and indels of the 991-accession collection, to identify SNPs associated with REL, MDA, POD, and SP under freezing stress [15,16]. The objective of this study was to identify the specific genomic regions and core genes that regulate freezing tolerance in rapeseed. The results revealed the identification of 1,158 SNPs related to freezing tolerance traits, which is crucial for elucidating the genetic architecture and pinpointing additional genes that underlie freezing tolerance in rapeseed. Meanwhile, specific genes under freezing stress by RNA-seq were used to narrow down the candidate genes. Ultimately, 73 genes were found to overlap between GWAS and RNA-seq results, and 13 candidate genes were identified as being involved in transmission and perception of freeze stress signals, lipid metabolism, ROS homeostasis, antifreeze protein synthesis, and other metabolic processes.

## Materials and methods

### Plant materials and growth conditions

The 289 core accessions of *Brassica napus* germplasm were selected from a worldwide germplasm collection of 991 accessions, and it is from Zhejiang University. The core accessions were sown and grown at the three experimental field locations, Tianshui of Gansu province (*34°.60′N,105°.64′E*), Jingchuan of Gansu province (*35°.37′N,107°.*20′*E*), and Yangling of Shaanxi province (*34°.27′N,108°.06′E*) to evaluate phenotypic values. Seeds were sown on August 19th in Jingchuan, on September 5th in Tianshui, and on September 20th in Yangling. When the minimum field temperature remained below -4 °C for three consecutive days, the third leaf was collected for phenotypic value measurement. Seedlings of genetic materials were harvested on November 25th in Jingchuan, on December 13th in Tianshui, and on December 21st in Yangling. The NS57, originating from the distant hybridization and cold acclimation between *Brassica napus* and *Brassica rapa*, has strong freezing tolerance in China’s northwestern areas. The NF24 is a normal spring rapeseed cultivar with a survival rate of 0% below -10°C. They are a typical combination for freezing tolerance research. The materials for RNA-seq (NF24 and NS57) were sown in pots (5L) filled with a 3:1 mixture of nutritional soil and vermiculite, and were grown in an illumination incubator under normal conditions (22/20°C, day/night temperature, 16 h/8 h light/dark cycle). When the seedlings of two cultivars reached the five-leaf stage, they were transferred to the chamber for freezing treatment at −4 °C for 12 h (Treatment 1, T1) and 24 h (Treatment 2, T2), while the control group (control, T0) was maintained in normal conditions. The third leaf was taken from the same position on each plant, immediately frozen in liquid nitrogen, and stored at -80 °C for subsequent RNA extraction.

### Phenotypic data collection

The REL was determined using a digital conductometer DDS-11A (Leici Instrument Factory, Shanghai, China) according to the method described by Wei et al. [17]. The MDA, POD, and SP were determined using an ultraviolet spectrophotometer Evolution 260 (Thermo Fisher Scientific, USA). The activities of POD were estimated following the procedures of Chen et al [18]. MDA and SP were measured according to the methods of Wei et al. [19] and Florencia et al. [20], respectively. REL was determined using fresh leaves, while other indicators were frozen and preserved in liquid nitrogen. The BLUE of each trait at three locations and replicates were calculated, and GWAS was performed.

### Genome-wide association study of four phenotypic values

The SNPs among the 289 accessions were downloaded from a website, which was identified in our previous study of the 991 accessions [16]. Using PLINK software with Minor Allele Frequency (MAF) > 0.05, genotyping rate (geno) <0.5 as the standard for quality control, 2,944,851 high-quality SNPs were identified in the 289 cultivars. Then, SNPs with uncertain chromosome locations in rapeseed were deleted, and a total of 2,284,466 SNPs were used for the GWAS. Principal component analysis (PCA) was performed using PLINK software. Admixture software was used to construct Q matrix of population structure. The K-value, representing the genetic relationships between samples, was calculated using Tassel 5.2 software. The Tassel 5.2 software with general linear model (GLM) and mixed linear model (MLM) was used for GWAS. In the MLM, Q- and K-values were used as fixed and random factors, while only Q-values were used in the GLM. After the analysis, the optimal model was selected based on the QQ plot for subsequent analysis. The p-value of each SNP was calculated, and a threshold of -log_1_P > 6 was defined as the suggestive. Meanwhile, when more than 30 significant loci were identified for a trait, the top 30 were selected to search neighboring genes. Then, 100kb sequence regions adjacent to the significantly associated SNPs were searched for the associate genes.

### KEGG enrichment and GO annotation analysis of neighboring genes

The neighboring genes identified from GWAS were annotated against Gene Ontology (GO) database and Kyoto Encyclopedia of Genes and Genomes (KEGG) database. The GO analysis was categorized into biological processes (BP), molecular functions (MF), and cellular components (CC).

### RNA-seq analysis and Crossover analysis with GWAS

Total RNA was extracted from treatment and control samples, each containing three biological replicates, was extracted using the TRIzol Reagent (Tiangen Biotech, China) according to the manufacturer’s instructions. The library construction and sequencing were performed by Gene Denovo Biotechnology Co. (Guangzhou, China) on the Illumina HiSeqTM 2500 platform. The reads containing adapters, reads containing poly-N, and low-quality reads from the raw data were removed using the Trimmomatic software. The short reads alignment tool Bowtie2 was used for mapping reads to the ribosome RNA (rRNA) database. The ribosome RNA (rRNA) mapped reads were removed [21]. Clean reads were subsequently mapped to the *Brassica napus reference genome* (Da-Ae) using TopHat2 software. The expression level was normalized by calculating the fragments per kilobase of exon model per million mapped fragments (FPKM) value. The DEGs between both samples were identified using the criteria of a *p*-value < 0.01 and |*log2 fold change*| ≥ 1. Local BLAST of protein sequences was used to convert IDs between different reference genomes. Then, the DEGs with significant differences between cultivars under freezing stress were used to narrow down the identified candidate genes.

### Transcription factors identified of neighboring genes

The *TF database of Brassica napus* was retrieved from the Plant TFDB v5.0 database. The expression data for the TFs were from the RNA-seq data. The genes were integrated into the R Programming language according to the IDs from the *Darmor database*.

## Result

### Genotype features of 289 genetic materials

**Fig 1:**
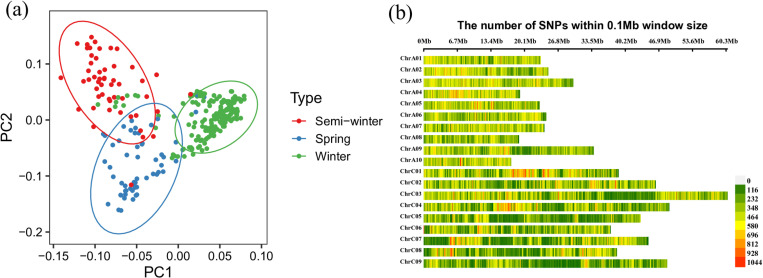
Genetic polymorphism of the 289 core accessions revealed by genome resequencing. (a) principal component analysis (PCA) plot of two components (PC1, PC2) using single-nucleotide polymorphisms (SNPs). (b) Single nucleotide polymorphism (SNP) density within a 0.1Mb window size on the 19 chromosomes of 289 resequenced genomes.

The 289 genetic materials from all over the world included 56 of spring rapeseed accessions, 53 semi-winter rapeseed accessions and 180 winter rapeseed accessions (S1 Table). Evidence from previous studies indicates that core-accessions population represent majority of the genetic diversity of the 991-accessions population. In this study, we re-filtered the resequencing data to obtain 2,944,851 high-quality SNPs. A PCA plot of the first two components (PC1 and PC2) of the 289 genetic materials is shown in Fig 1a, and three different types of rapeseeds are grouped separately. After removing SNPs with uncertain chromosomal locations, 2,284,466 SNPs were distributed across the 19 rapeseed chromosomes (Fig 1b). The number of SNPs per chromosomes of C03 is largest, and lowest for A08. The average number of SNPs per 1 kb of chromosomes was more than 2.46, and with the highest density observed on chromosome A04 (S1 Fig).

### Phenotypic features of the indices related to cold tolerance

**Fig 2:**
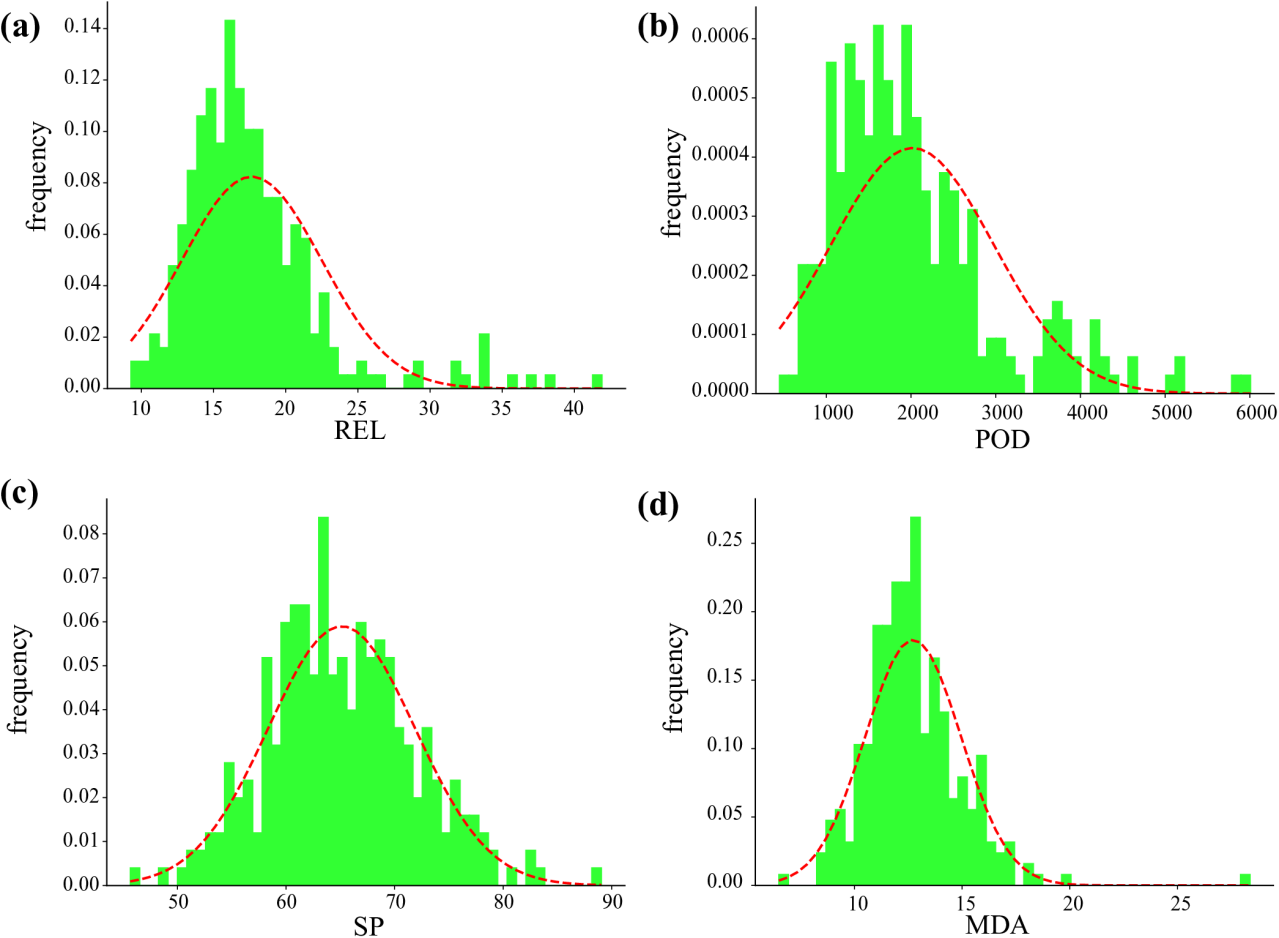
The frequency distributions map of phenotypic BLUE values in 289 core accessions at different locations. (a) The BLUE value of REL. (b) The BLUE value of POD. (c) The BLUE value of SP. (d) The BLUE value of MDA.

**Fig 3:**
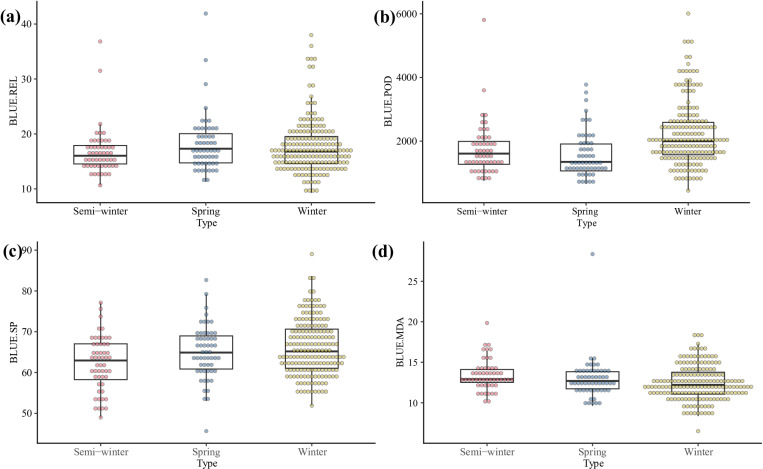
Box plot of phenotypic BLUE values in 289 core accessions at different locations. (a) The BLUE value of REL.(b) The BLUE value of POD. (c)The BLUE value of SP. (d) The BLUE value of MDA.

When the minimum field temperature was below −4 °C and lasted for three days, phenotypic values were obtained from the core-accessions. Meanwhile, the BLUE for each trait was calculated with the locations, replicates as random factors and genotypes as fixed factors. The frequency distributions of phenotypic values and BLUE values for each trait conform to normal or approximately normal distributions (Fig 2a-d and S2 Fig). The phenotypic values of three biotypes showed obvious differences, and the MDA and REL values of winter rapeseed seedlings were significantly lower than those of semi-winter/spring rapeseeds, while the values of POD and SP were higher (Fig 3a-d and S3 Fig). The correlation coefficients (r) of the four phenotypes at the three experimental locations were provided in S2 Table. Overall, MDA exhibited a positive correlation with REL, while SP showed a positive correlation with POD. Additionally, MDA and REL were negatively correlated with both SP and POD.

### Genome-wide association analysis of freezing stress-related indices

**Fig 4:**
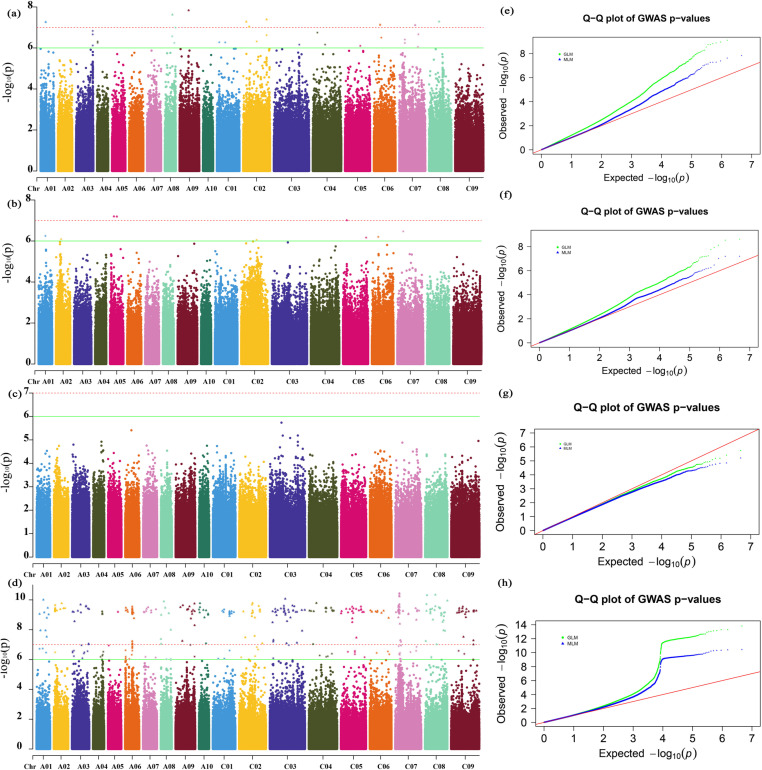
Manhattan plots and QQ plots of GWAS for BLUE. (a, e) the BLUE of REL among three locations. (b, f) the BLUE of POD among three locations. (c, g) the BLUE of SP among three locations. (d, h) the BLUE of MDA among three locations.

**Fig 5:**
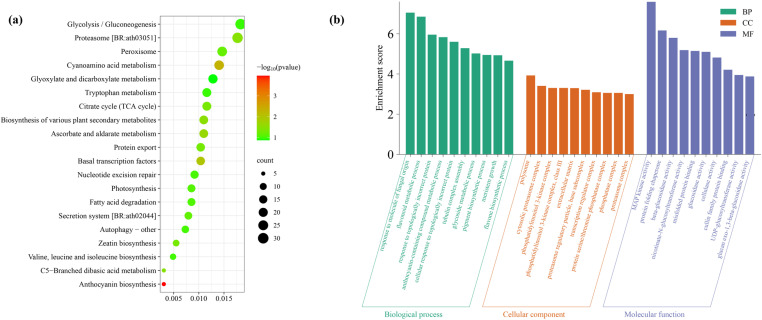
The pathway enriched of 4,998 neighboring genes. (a) the KEGG pathways of neighboring genes. (b) the GO classification annotation of neighboring genes.

GWAS were performed for the four phenotypic traits of 289 core accessions in three locations and BLUE values. In total, 594, 513, 7, and 45 SNPs were significantly associated with MDA, POD, SP and REL values (Fig 4 and S4 Fig). Based on these significantly associated SNPs, we identified 4,998 associated genes (S3 Table). KEGG enrichment analysis showed that these genes were involved in anthocyanin biosynthesis, cyanoamino acid metabolism, basal transcription factors, ascorbate and aldarate metabolism, biosynthesis of various plant secondary metabolites, etc (Fig 5a). GO classification annotation found that these genes were annotated to 101 functional categories, including 79 biological processes, 11 molecular functions, and 11 cellular components (Fig 5b). Among these biological processes, response to molecules of fungal origin, flavonoid metabolic process, topologically incorrect protein, anthocyanin-containing compound metabolic process were the most significant. MAP kinase activity, protein folding chaperone, beta-glucosidase activity, and glucosidase activity were the most represented GO categories in the molecular functions. At the same time, the polysome, cytosolic proteasome complex, and phosphatidylinositol 3-kinase complex were the top three significantly dominant cellular components.

### Differences in gene expression between two cultivars

**Fig 6:**
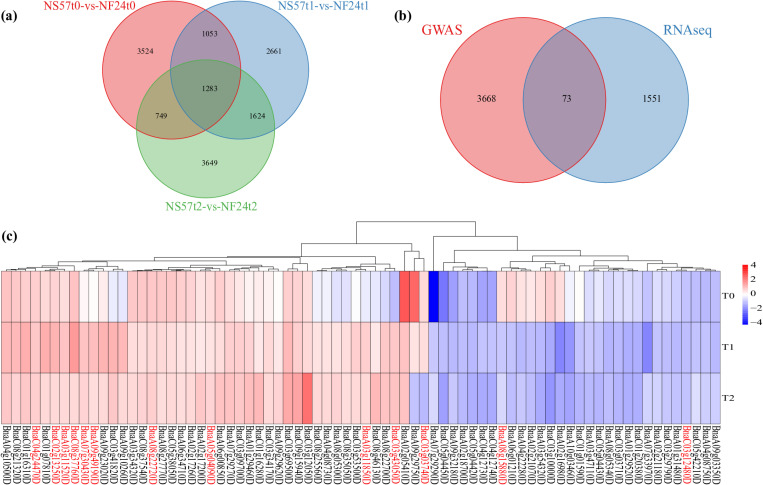
Differentially expressed genes (DEGs) in the freezing-sensitive and freezing-resistant cultivars revealed by RNA-seq analysis. (a) the Venn plot of DEGs under freezing stress treatment. (b) the Venn plot of genes between GWAS and RNA-seq. (c) the Heat map of overlapped genes between GWAS and RNA-seq.

In order to test transcription-level changes, a pair of freezing-sensitive and freezing-resistant cultivars, NF24 and NS57, were used for transcriptome sequencing, consisting of three replicates and three treatments. The high-quality clean reads per library were exceeded 39 million, the Q30 for all libraries was greater than 94%, and the GC content per library was approximately 48%. For all libraries, over 77% of reads were uniquely mapped to the reference genome and the mapping ratio was close to 80%. At the normal temperature, 6,609 DEGs were identified between two cultivars, including 3,840 upregulated and 2,869 downregulated DEGs. After T1 freezing stress, 6,621 DEGs were found to be accumulated between the two cultivars, including 4,100 upregulated and 2,521 downregulated DEGs. Meanwhile, 7,305 DEGs were identified between the two cultivars under T2 freezing stress, including 3,692 upregulated and 3,613 downregulated DEGs. Finally, 1,624 DEGs were common to both T1 and T2 stress, which were specific to freezing stress. These genes likely from the basis for freezing tolerance variation in rapeseed (Fig 6a). Therefore, these 1,624 genes utilized for further intersection analysis between the candidates identified through GWAS and RNA-seq.

### Crossover analysis between the candidates identified by GWAS and RNA-seq

RNA-seq analysis was used to narrow down the candidate genes identified by GWAS, which helps to improve the efficiency and accuracy of identification. After consistent reference genomes, the 3,730 genes were successfully converted from 4,998 neighboring genes, corresponding to 3,741 unique genes. The crossover analysis showed that 73 genes were overlapping (Fig 6b). The IDs of the candidate genes and their annotations are listed in (Table 1) S1 Table. And among these, 13 of these genes are more closely related to freezing stress and are potential candidates for further research. They are involved in the transmission and perception of freezing stress signals, lipid metabolism, ROS homeostasis, antifreeze protein synthesis, and other metabolic processes.

**Table 1:** **Candidate genes narrowed down by GWAS and RNA-seq experiments and their annotations**.

Name	Gene ID	annotation
**BnADH**	BnaC04g24470D	alcohol dehydrogenase-like
**BnCaM**	BnaC03g43050D	calmodulin-like protein 11, partial
**BnCML**	BnaA08g22720D	probable calcium-binding protein CML27
**BnAFP12**	BnaA03g11520D	glucan endo-1,3-beta-glucosidase 12-like isoform X1
**BnAFP13**	BnaC02g13250D	glucan endo-1,3-beta-glucosidase 13-like
**BnAPX**	BnaA09g49190D	L-ascorbate peroxidase 1, cytosolic
**BnLOX**	BnaC08g37760D	lipoxygenase 3, chloroplastic
**BnFQR2**	BnaA05g04090D	NADH dehydrogenase ubiquinone 1 alpha subcomplex assembly factor-like protein
**BnFQR1**	BnaA03g11850D	NAD(P)H dehydrogenase (quinone) FQR1
**BnNQO-M**	BnaA08g15880D	NAD(P)H-quinone oxidoreductase subunit M, chloroplastic
**BnNQO-N**	BnaC03g12480D	NAD(P)H-quinone oxidoreductase subunit N, chloroplastic
**BnPP2C**	BnaA07g30430D	protein phosphatase 2C 16-like
**BnATAF2**	BnaC03g03740D	protein ATAF2-like

### Transcription factors identified for neighboring genes

**Fig 7:**
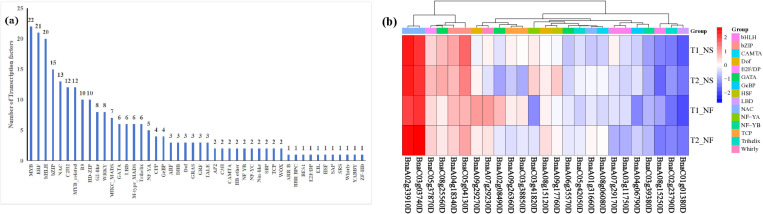
The transcription factors identified of neighboring genes. (a)Total number of transcription factors in neighboring genes. (b) Number of transcription factors identified in both cultivar after freezing stress.

The neighboring genes were annotated to transcription factors database to further understand the function. The result showed that 268 non-redundant TFs were predicted, which belonged to 46 TF families (Fig 7a). The MYB (34 members) was the TF family with the most members, including 22 MYB and 12 MYB-related members, and ERF was the second-largest TF family with 21 members. The bHLH was in third place (20 members), followed by the bZIP(15), NAC (13), and C2H2 (12). Among these TFs, only 36 TFs were expressed with expression in FPKM value ≥1. The expression levels of 25 TF genes significantly altered under freezing stress, whereas the expression of 8 TF genes remained consistent between two varieties (Fig 7b). These transcription factors may play a crucial role in *Brassica napus* freezing tolerance, including bZIP, bHLH, NAC, Trihelix, NF-YB, and LBD. The bZIP, NAC were upregulated under freezing stress, while the other TFs were downregulated.

## Discussion

Winter freezing stress is one of the key limiting factors for rapeseed cultivation in northwest China [22]. The scarcity of freezing-tolerant genetic resources and cultivars led to winter rapeseed difficult to overwinter in these areas, thereby restricting its further northern expansion of winter rapeseed growing areas [3]. Thus, the identification and utilization of novel genes and genetic resources associated with freezing tolerance is important for improving the freezing tolerance of winter rapeseed. This can accelerate the northward migration of *brassica napus* to replace the winter turnip rape (*Brassica rapa*) with strong freezing tolerance and low quality and yield. A substantial body of evidence indicates that freezing stress damages cell membranes, as ice nuclei form intracellularly at sub-zero temperatures, leading to cellular water loss, extracellular leakage of intracellular substances, and an increase in relative electrolyte leakage (REL) [23]. In the present study, we found that REL was negatively correlated with freezing tolerance, and the REL of spring rape reached its highest level following freezing stress. Additionally, lipid, which are the primary component of cell membranes, are oxidized to malondialdehyde (MDA) under various stress conditions [13]. Furthermore, peroxidase (POD), an oxidoreductase in plants, plays a crucial role in reduction reaction of H_2_O_2_ [24]. Soluble protein (SP) enhance plant intracellular osmosis, thereby inhibiting the formation of initial ice nuclei under freezing stress [25].These studies have been demonstrated that freezing-tolerant cultivars exhibit higher levels of POD and SP under freezing stress, while MDA levels are lower [2,17].

GWAS is an efficient strategy to identify functional genes controlling agronomic or quality traits, which is widely used to locate plant stress-related genes [26–28]. Under drought stress, GWAS identified 1,281 SNPs associated with agronomy and yield-related traits in rapeseed, leading to the identification of 2,232 candidate genes within their linkage disequilibrium (LD) regions [29]. Analysis of another GWAS revealed the identification of 60 candidate genes related to drought stress, among which 18 were involved in phenylpropanoid pathway and flavonoid modifications, including *phenylalanine ammonia-lyase 1-like (PAL1)*, *chalcone-flavanone isomerase (CHI)*, *flavonol 3-O-glucosyltransferase (UGT89B1)*, *flavonol synthase3 (FLS3)*, *cinnamoyl-CoA reductase 1 (CCR1)*, and *flavonoid 3’-monooxygenase (CYP75B137)* [30]. In freeze stress studies, the chlorophyll fluorescence and average damage index of 399 canola cultivars frozen at -15°C for 3 days and 7 days were used as traits for GWAS analysis [10]. This resulted in the identification of 13 SNP loci related to the cold tolerance, with 25 candidate genes screened within their LD intervals, primarily involved in physiological and molecular processes such as plant photosystem protection, cell membrane stability, and RNA integrity. In the same year, another GWAS study on 222 canola cultivars identified 13 SNPs and 24 candidate genes associated with freezing tolerance in rapeseed [9]. Functional annotation indicated that these genes encoded proteins related to lipid and fatty acid metabolism, photosynthesis, flowering, ubiquitination and cytochrome P450. In contrast, our 289 cultivars contained more SNP loci, which would greatly improve the efficiency of GWAS detection. Our study uncovered 1,158 SNPs across nineteen chromosomes correlated with variation in freezing stress-related indices through GWAS analysis. Additionally, we identified 4,998 neighboring genes located within 100 kb upstream or downstream of these partially significant SNP (Fig 4 and S4 Fig).

The KEGG analysis revealed that these genes were involved in anthocyanin biosynthesis, cyanoamino acid metabolism, basal transcription factors, ascorbate and aldarate metabolism, as well as the biosynthesis of various plant secondary metabolites (Fig 5). Previous studies have shown that these metabolic pathways have various important roles in plants, including production, development, and adaptation to stressful environments [30–32]. Analysis of transcription factors found that bZIP, bHLH, NAC, Trihelix, NF-YB, LBD are the predominant families among neighboring genes. Notably, all members of the bZIP and NAC families were upregulated in response to freezing stress (Fig 6). The plant-specific NAC proteins represent one of the largest TF families, which play a crucial role in regulating the expression of the structural genes involved in stress response pathways [33,34]. Studies on purple Chinese cabbages have demonstrated that low temperature promotes the biosynthesis of anthocyanins and the expression of related genes in seedlings [35]. Under drought stress, the transcription of NAC035 in rapeseed was upregulated, particularly in high-phenolic genotype [34]. The ATAF2 belongs to NAC transcription factor family in Arabidopsis and is known to regulate responses to biotic stress [36]. In this study, BnaC03g03740D encodes *BnATAF2*, and its expression increased by more than 4 times in both rapeseed cultivars after freezing stress. The transcription factor of bZIP is associated with a wide range of abiotic stresses [37]. In wheat, the research has demonstrated that *TabZIP15* improves salt stress tolerance. In rice, *OsbZIP16* and *OsbZIP62* positively regulate the drought tolerance [38]. In potatoes, overexpression of the bZIP transcription factor *StbZIP-65* enhances salt stress tolerance [39]. In plum blossoms, *PmbZIP12/31/36/41/48* all respond to low-temperature stress. Notably, *PmbZIP31/36/41* exhibit more prominent response to freezing stress compared to cold level, as they are almost entirely upregulated freezing treatment [40]. The findings indicated that these transcription factors are closely related to freezing tolerance of rapeseed under freezing stress.

Linkage mapping and genome-wide association studies are important and effective methods for gene identification, especially in rice and Arabidopsis [36,41,42]. Hang Zheng et al. conducted a QTL analysis of the cold tolerance in *Brassica napus* using F2:3 populations [43]. They identified 11 QTLs associated with five phenotypic traits related to cold tolerance, distributed across six linkage groups, including six major QTLs that account for more than 10% of the phenotypic variation. In a subsequent study, 11 QTLs related to electrolytic leakage (REL) and overwintering rate (OER) were found in another F2:3 population. Notably, two major QTL explained more than 10% of the phenotypic variance were limited into the same region from 15.43 cM to 32.76 cM on chromosome A07 [6]. Meanwhile, the gene encoding β-1, 3-glucanase protein positively regulates freezing tolerance of rapeseed within this region [7]. In this study, two orthologous genes were identified, both of which exhibited induced expression under freezing stress. Notably, the expression levels in freezing-resistant cultivars were significantly higher than those in freezing-sensitive cultivars. In the GWAS of Danielle F. Wrucke, the one QTL that explained about 5% of the phenotypic variation and located on chromosome C04 was identified associated with frost tolerance of canola, which contains the gene encoding protein phosphatase 2C (PP2C) [11]. *PP2C*, as a negative regulator of serine/threonine protein phosphatase, plays an important role in abscisic acid (ABA) and abiotic-stress-mediated signaling pathways in plant [44].In wheat, phenotypic analysis of overexpression and CRASP/Cas9 mutant lines indicated that *TaPP2C158* was a negative regulator responsible for drought resistance [45]. However, genome-wide identification of the PP2C gene family of wild sugarcane showed that a total of 27 genes were induced expressions by cold stress [46]. Therefore, it is plausible that *PP2C* may exhibit diverse regulatory mechanisms in response to different types of stress. BnaA07g30430D, a putative orthologs of *PP2C*, exhibited higher expression levels in the freezing-tolerance cultivars compared to freezing-sensitive ones, implying that the *BnPP2C* may function as a positive regulator of freezing stress responses in this context.

The balance of reactive oxygen species (ROS) is broken under freezing stress, leading to the activation of metabolic pathways and genes related to plant oxidative stress [47]. NAD(P)H dehydrogenase functions in alleviation of oxidative damage caused by temperature stress [48]. Following freezing stress, the expression levels of two homologs encoding NAD(P)H dehydrogenase increased, with significantly higher expression observed in freezing-tolerance cultivars compared to freezing-sensitive ones. Similarly, a gene encoding alcohol dehydrogenase exhibited an expression pattern consistent with the aforementioned NAD(P)H dehydrogenase genes. In contrast, the expression levels of two genes encoding NAD(P)H-quinone oxidoreductase subunits M/N decreased after expose to freezing stress. *Ascorbate peroxidase* (*APX*) is a crucial antioxidant enzyme that plays a significant role in H_2_O_2_ scavenging system of the ascorbate-glutathione cycle in plant cells [49]. In eggplants, members of the APX family have shown responses to high temperatures, especially *SmAPX2* [50]. Silencing *SmAPX2* has resulted in a notable reduction in stress resistance. It has been proved that *APX* is closely related to the abiotic stress of *Brassica rapa* [51]. In this study, a gene encoding *BnAPX* was also detected, and its expression increased more than twofold in both cultivars under freezing stress. Notably, the expression level was higher than in freezing-tolerance cultivar compared to the freezing-sensitive cultivar.

In response cold stress, Ca^2+^ plays an important role in the sensing and transmission of stress signals, and calmodulin and *calcium-binding protein CML27* are key components of calcium signaling pathway [52]. The RNA-seq data showed that their expression levels in the winter rapeseed leaves increased 1–3 fold after freezing stress, with a more pronounced increase observed in freezing-resistant cultivars. *Lipoxygenase* (*LOX*) is a non-heme iron-containing dioxygenase family that catalyzes the oxygenation of polyunsaturated fatty acids into bio-functionally acid diverse (oxylipins) and plays a vital role in plant growth, development, and responses to abiotic stress [53]. The RNA-seq data showed that it was upregulated expression more than sixfold in both cultivars 24 hours after freezing stress. In addition to the above genes, we also detected other related genes, but the relationship between these genes and freezing stress response in rapeseed needs further investigation and analysis.

## Conclusion

In summary, a total of 1,158 SNPs and 4,998 neighboring genes were identified across nineteen chromosomes. Crossover analysis between the candidates identified by GWAS and RNA-seq identified 73 overlapping genes. Combined with gene annotation and gene expression data, 13 genes were determined to be more closely related to freezing tolerance in rapeseed. These data can provide new genes resource for further investigation into the mechanism of freezing tolerance in rapeseed.

## Supporting information

S1 Fig The average number of SNPs per 1 kb and number of SNPs per chromosome. (TIF)

S2 Fig Frequency distributions of four traits in 289 core accessions. (a-d) Frequency distributions of REL, POD, SP, and MDA in Jingchuan location. (e-h) Frequency distributions of REL, POD, SP, and MDA in Tianshui location. (i-l) Frequency distributions of REL, POD, SP, and MDA in Yangling location.
(TIF)

S3 Fig Box plot of four traits in 289 core accessions. (a-d) Box plot of REL, POD, SP, and MDA in Jingchuang location. (e-h) Box plot of REL, POD, SP, and MDA in Tianshui location. (i-l) Box plot of REL, POD, SP, and MDA in Yangling location.
(TIF)

S4 Fig Manhattan plots of GWAS for four phenotypes value. (a-d) Manhattan plot of REL, POD, SP, and MDA in Jingchuang location. (e-h) Manhattan plot of REL, POD, SP, and MDA in Tianshui location. (i-l) Manhattan plot of REL, POD, SP, and MDA in Yangling location.
(TIF)

S1 Table Source information for 289 genetic materials. (XLSX)

S2 Table The correlation coefficients of the REL, POD, SP, and MDA at the three locations and BLUE value. (XLSX)

S3 Table The genes were associated with significantly SNPs. (XLSX)

S1 File Assign automatically. (DOCX)
