# Supplementary figures and images for: Genome-wide association studies and transcriptome analysis reveal novel genes associated with freezing tolerance in rapeseed (Brassica napus L.)
Source: PLoS One. 2025 May 27;20(5):e0322547. doi: 10.1371/journal.pone.0322547 (PMC12111304; doi:10.1371/journal.pone.0322547)

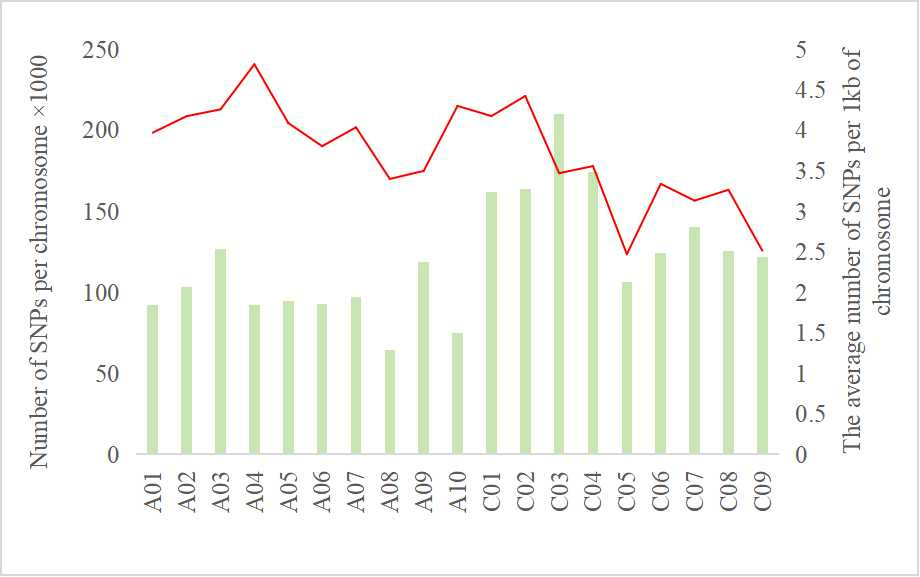

Supplement: S1 Fig — (TIF) [file pone.0322547.s001.tif]

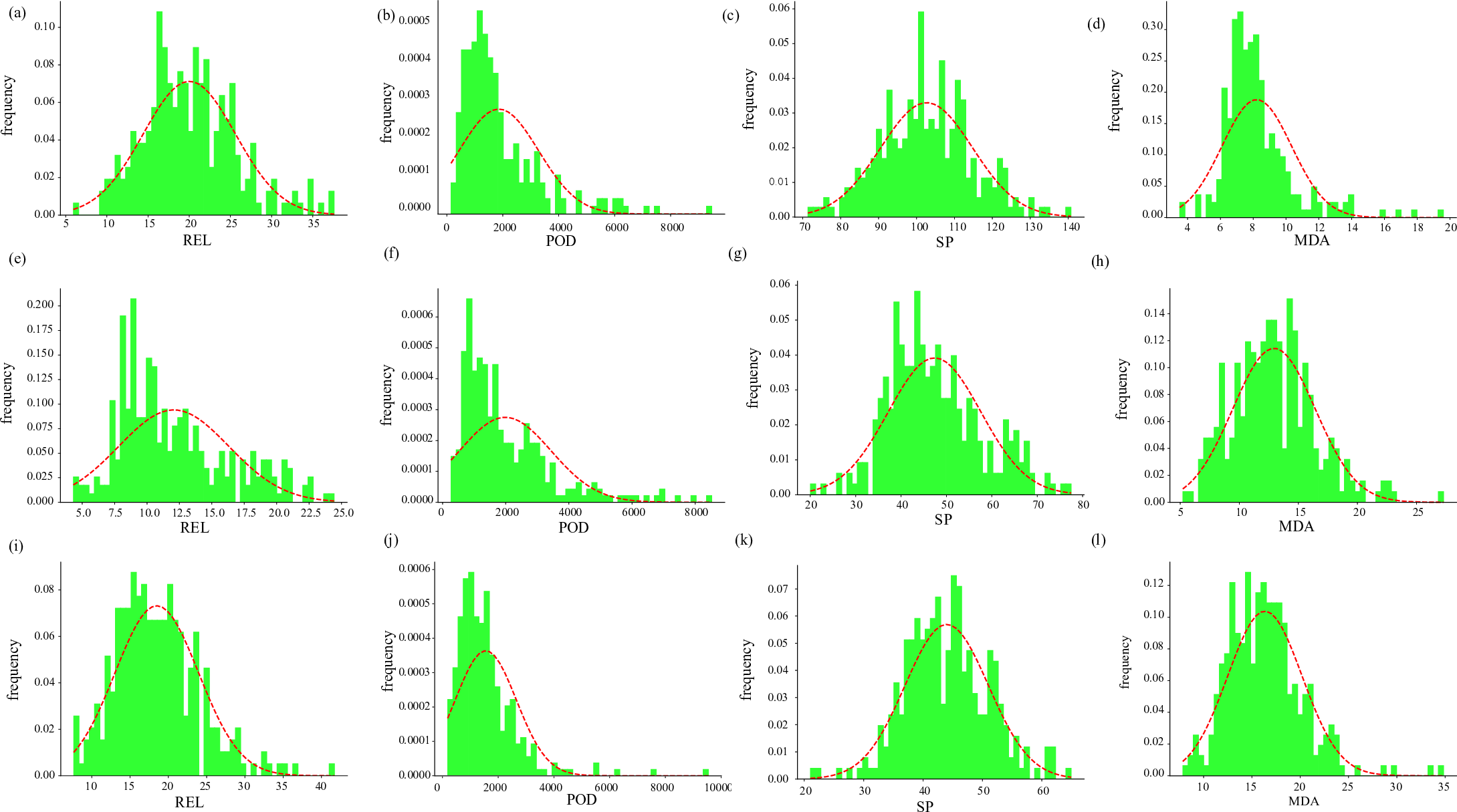

Supplement: S2 Fig — (a-d) Frequency distributions of REL, POD, SP, and MDA in Jingchuan location. (e-h) Frequency distributions of REL, POD, SP, and MDA in Tianshui location. (i-l) Frequency distributions of REL, POD, SP, and MDA in Yangling location. (TIF) [file pone.0322547.s002.tif]

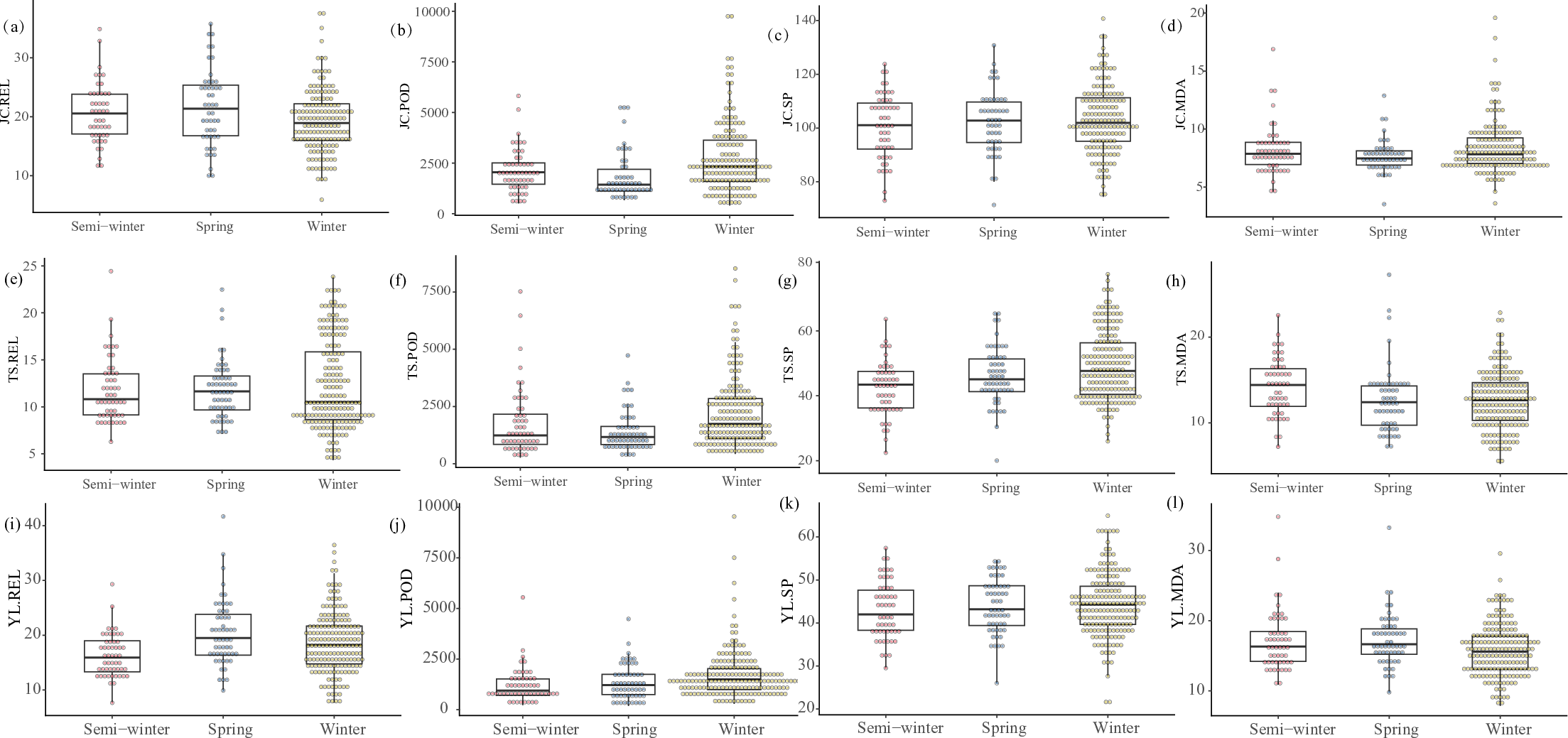

Supplement: S3 Fig — (a-d) Box plot of REL, POD, SP, and MDA in Jingchuang location. (e-h) Box plot of REL, POD, SP, and MDA in Tianshui location. (i-l) Box plot of REL, POD, SP, and MDA in Yangling location. (TIF) [file pone.0322547.s003.tif]

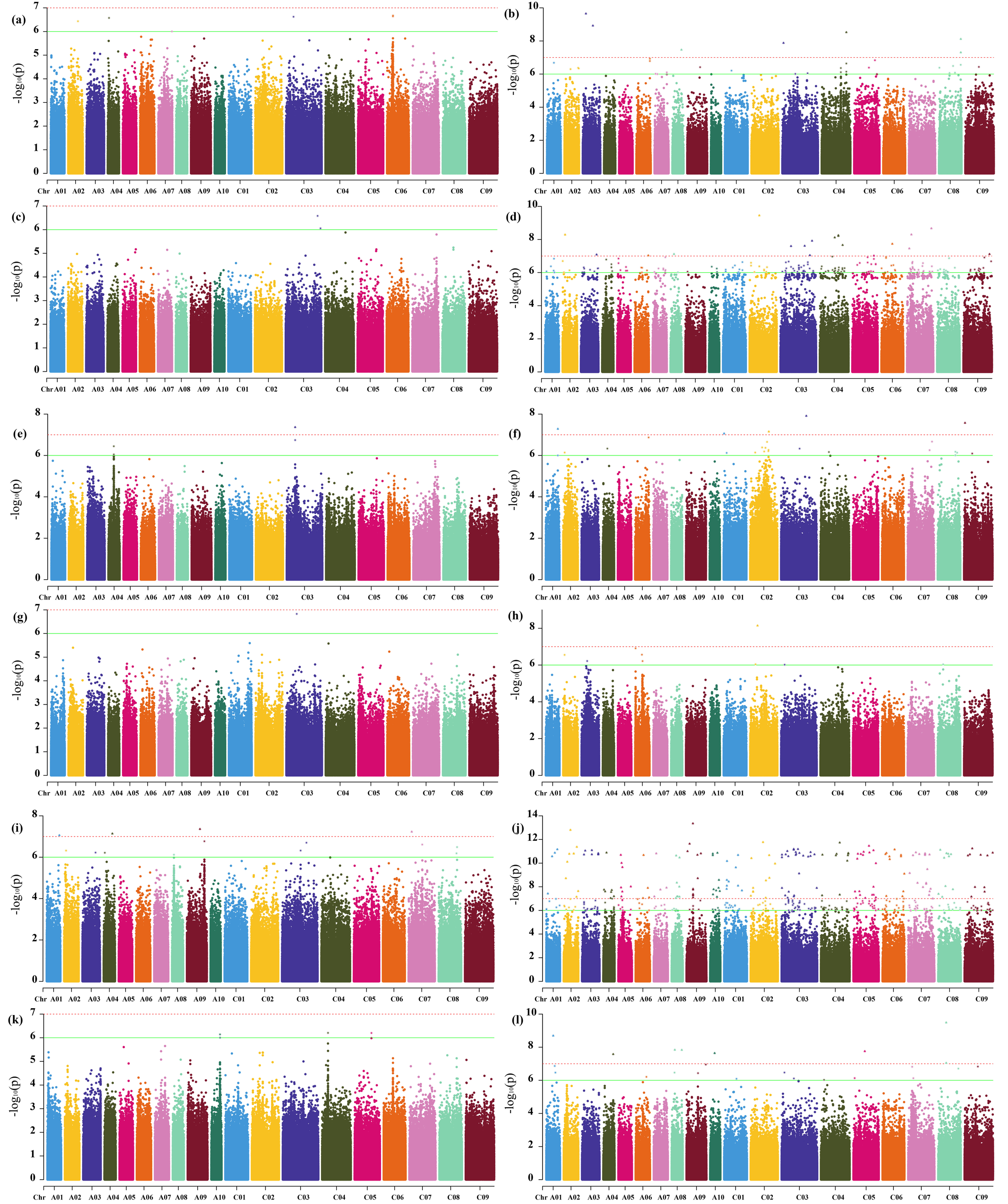

Supplement: S4 Fig — (a-d) Manhattan plot of REL, POD, SP, and MDA in Jingchuang location. (e-h) Manhattan plot of REL, POD, SP, and MDA in Tianshui location. (i-l) Manhattan plot of REL, POD, SP, and MDA in Yangling location. (TIF) [file pone.0322547.s004.tif]
