# Supplementary material for: Genome-wide association studies and transcriptome analysis reveal novel genes associated with freezing tolerance in rapeseed (Brassica napus L.)
Source: PLoS One. 2025 May 27;20(5):e0322547. doi: 10.1371/journal.pone.0322547 (PMC12111304; doi:10.1371/journal.pone.0322547)
Supplement: S1 File — (DOCX) [file pone.0322547.s008.docx]

The author list：

Zigang Liu:

Conceptualization

Resources

Guoqiang Zheng:

Conceptualization

Formal analysis

Investigation

Software

Visualization

Writing – original draft

Lixi Jiang:

Resources

Qi Yang:

Formal analysis

Writing – review & editing

Jiaping Wei:

Project administration

Writing – review & editing

Zefeng Wu:

Methodology

Writing – review & editing

Junmei Cui:

Supervision

Writing – review & editing

Xiaoyun Dong:

Investigation

Xiaodong Cao:

Data curation

Xuezhen Yang:

Investigation

Ying Wang:

Investigation

Yongjie Gong:

Investigation

Ermei Sa:

Investigation

Xiaoxia Wang:

Investigation

Competing Interests: The authors have declared that no competing interests exist.

Financial Disclosure: Natural Science Foundation of China [32360520], the Natural Science Foundation of Gansu Province [23JRRA1408],Gansu Province Science and Technology Major Project [22ZD6NA009], scientific and technological projects of Gansu Province [23CXJA0001], the Key Talent Projects of Gansu Province [2023RCXM33], the scientific and technological projects of Linzhi City [LZZX-04], and the Innovator’s Star project [2025CXZX].The funders had no role in study design, data collection and analysis, decision to publish, or preparation of the manuscript.
